# New Department for Military Orthopedics Organized by Surgeon General of the Army

**Published:** 1917-08

**Authors:** 


### NEW DEPARTMENT FOR MILITARY ORTHOPEDICS ORGANIZED BY SURGEON GENERAL OF THE ARMY.

### Special Section to Deal With Cases Both at Home and Abroad—Maj. Elliott Brackett Appointed Director.

The Surgeon General has made the following announcement of the organization of a new department for military orthopedics:

The very large percentage of the casualties of the present war which require special orthopedic methods in their treatment (from 30—40 per cent.), and the large percentage of these cases, when so treated, that can be restored to military usefulness (from 70—75 per cent.), has led the Surgeon General to create an organization to care for these cases. This will be designated the department for military orthopedics and will have to do with the work that is required both at home and abroad.

### Maj. Brackett, Director.

Maj. Elliott G. Brackett, Medical Reserve Corps, has been appointed director of military orthopedics, with headquarters at the Surgeon General's Office.

Maj. David Silver, Medical Reserve Corps, has been appointed assistant director of military orthopedics, with the same headquarters as the director.

For the expeditionary forces, while the work will be under the authority of the director, nevertheless so much special organization will be required that the office of director of military orthopedics for the expeditionary forces has been created, and Maj. Joel E. Goldthwait, Medical Reserve Corps, has been appointed to fill that position.

Associated with him and to serve as assistant directors, Maj. Robert B. Asgood, now serving with the United States Base Hospital No. 5, and Capt. Nathaniel Allison, now serving with the United States Base Hospital No. 21, will be transferred from their present positions to this department. Maj. Osgood will be temporarily assigned to Col. Robert Jones, the director of military orthopedics for the British forces, for the study of details of organization and methods of treatment, and Capt. Allison will be temporarily assigned to similar study with the French and Italian Forces.

For the assistance of the directors, an advisory orthopedic board has been created and is made up as follows: Dr. Robert W. Lovett, Boston, Mass.; Dr. Albert H. Freiberg, Cincinnati, Ohio; Dr. G. Gwillym Davis, Philadelphia, Pa.; Dr. F. H. Alber, New York, N. Y.; and Dr. John L. Porter, Chicago, Ill.

The classifications adopted, of the conditions to be considered orthopedic, is practically the same as that in use by the British Government, and is as follows:

a. Derangements and disabilities of joints, including anchylosis.

b. Deformities and Disabilities of the feet, such as hallux valgus, hallux rigidus, hammer toes, metatarsalgia, painful heels, flat or claw feet.

c. Malunited or ununited fractures.

d. Injuries to ligaments, muscles, and tendons.

e. Cases requiring tendon transplantations or other treatment for irreparable destruction of nerves.

f. Nerve injuries, complicated with fractures or stiffness of joints.

g. Cases requiring surgical appliances, including artificial limbs.

